# Triplane Fracture of the Proximal Tibia

**DOI:** 10.5435/JAAOSGlobal-D-21-00040

**Published:** 2021-07-07

**Authors:** Austin Whiting, Manaf Younis, Lauren Saunee, Carter Clement

**Affiliations:** From the Ascension Genesys, Grand Blanc Township, MI (Dr. Whiting); and Childrens Hospital New Orleans, New Orleans, LA (Dr. Younis, Saunee, Dr. Clement).

Triplane fracture of the distal tibia is a well-known injury that was first described by Lynn^1^ in 1972. Triplane fractures cross through the articular surface, physis, and metaphysis in the sagittal, transverse, and coronal planes, respectively. Triplane fractures of the proximal tibia in skeletally immature patients are rare injuries with only a handful reported in the literature to our knowledge.^2,3^ We present the case of a 13-year-old boy who sustained a proximal tibial triplane fracture and subsequently underwent arthroscopic-assisted reduction and internal fixation.

## Case Report

**Figure 1:**
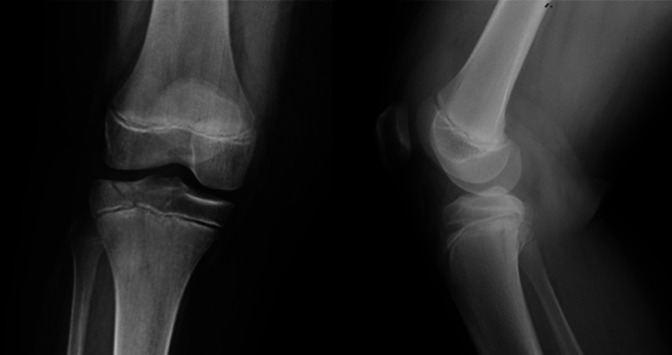
Radiographs of the knee showed a Salter-Harris 4 fracture of the proximal tibia with 4-mm displacement of the epiphysis in the coronal plane and a minimally displaced posterior metaphyseal fracture in the sagittal plane.

**Figure 2:**
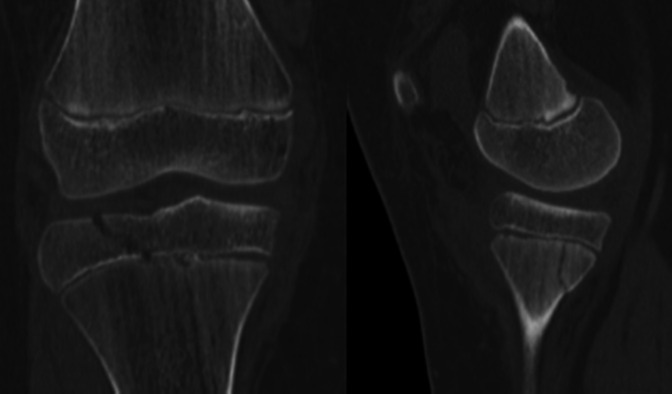
CT scan further defining the injury.

A 13-year-old boy presented to the emergency department after falling and twisting his knee at a trampoline park. On physical examination, he had a tense knee effusion and painful range of motion and was otherwise neurovascular intact without any signs of compartment syndrome. Radiographs of the knee showed a Salter-Harris 4 fracture of the proximal tibia with 4-mm displacement of the epiphysis in the sagittal plane and a minimally displaced posterior metaphyseal fracture in the coronal plane (Figure 1). He also had an associated proximal fibula fracture. The fracture was better defined on CT demonstrating the coronal, sagittal, and axial components of his injury (Figure 2).

**Figure 3:**
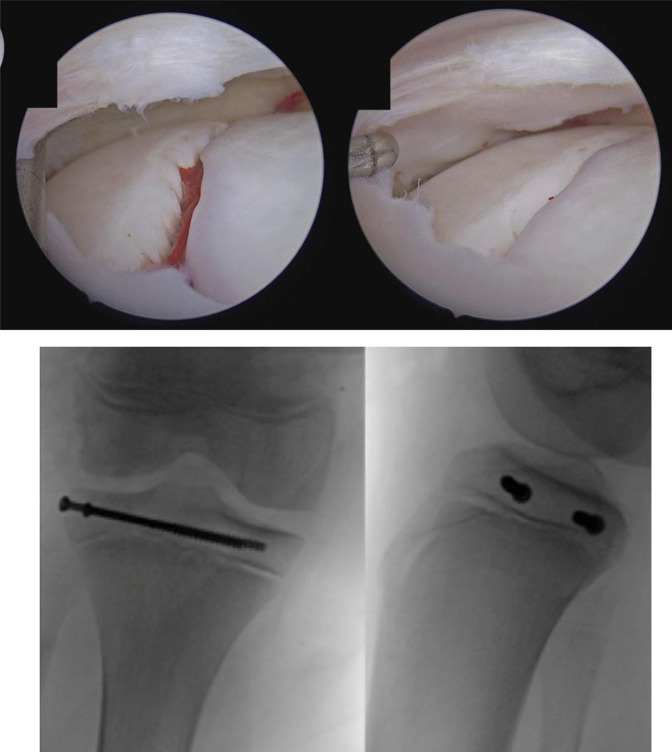
Intraoperative fluoroscopy shows anatomic reduction of the fracture. Pre and post reduction arthroscopy of the fracture allowed for direct visualization of the mandatory reduction of the articular surface.

**Figure 4:**
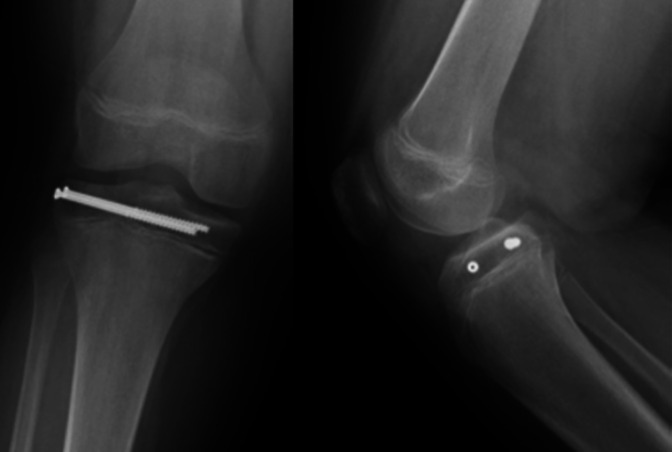
Six-week radiographs show healing of the fractures in the anatomic position.

Under general anesthetic, the patient was positioned in the supine position with the thigh in a leg holder, and the foot of the table was dropped. A standard high lateral arthroscopy portal was created, and a diagnostic examination was performed. A large hemarthrosis was initially noted. A shaver was introduced through a medial portal, and after extensive débridement, adequate visualization was achieved. Mild fraying of the white-white zone of the body of the lateral meniscus was noted as well as the fracture line of the tibia in the lateral compartment with 4 mm of gapping and interposed hematoma. There were no other injuries noted in the knee. The frayed meniscus was débrided. Under fluoroscopy, two guidewires for 4.0-mm cannulated screws were inserted from the lateral tibial plateau across the epiphysis, roughly perpendicular to the fracture line. The outer cortex was overdrilled, and two partially threaded 60-mm cannulated screws were advanced over the pins. Orthogonal fluoroscopy demonstrated appropriate transepiphyseal screw position, and the fracture was appropriately reduced under arthroscopic visualization (Figure 3). Postoperatively, the patient was discharged home in a knee immobilizer with instructions for 6 weeks of non–weight bearing on the extremity. At his 6-week follow-up, the fracture was healing in the anatomic position (Figure 4), and he was transitioned to full weight bearing with instructions to avoid sports and other rigorous activities for another 4 weeks. Plans were made to see him back at 6 to 12 months to rule out growth disturbance and to discuss the pros and cons of screw removal.

## Discussion

Triplane fractures typically occur in the distal tibia but can also be seen in the proximal tibia. It has been proposed that triplane fractures of the proximal tibia are relatively rare because of the symmetrical closure pattern of the physis in contrast to the asymmetrical closure of the distal tibial physis.^2,4,5^ It has also been suggested that the medial and lateral collateral ligaments of the knee pass over the epiphysis and insert onto the tibial metaphysis, which protects the epiphysis from fractures by allowing stress to bypass the epiphysis, being transmitted directly to the metaphysis.^5^

The primary treatment goal for this injury is anatomic reduction of the articular surface with avoidance of further injury to the physis. Arthroscopy can be valuable to assess injury to other intra-articular structures such as the cruciate ligaments and menisci, to directly visualize the articular surface reduction, and to completely evacuate the fracture hemarthrosis.^2,6^ If arthroscopy is not available to directly visualize the tibial epiphyseal cartilage, arthrography can also be used. In our case, arthroscopy allowed diagnosis and treatment of a minor meniscal injury. In addition, although the fracture was visible on radiographs, CT was integral in obtaining a complete understanding of the triplane nature of this unusual pattern.^3,7-9^ If additional injury to intra-articular structures is suspected, an MRI could also provide information to guide surgical treatment.

A learning point from our case was related to the size of the cannulated screws used for fixation. Both 4.0- and 4.5-mm diameter screws were considered, and the former was chosen to minimize the risk of iatrogenic damage to the physis or articular surface during placement. Although reduction was successful, the bite of the screws was not excellent, and the screw heads began to sink into the soft metaphyseal cortex. In future cases, we will use 4.5-mm screws because the additional 0.5-mm diameter can be safely accommodated by the epiphysis with careful screw positioning. In addition, using a washer will also be considered. The increased screw diameter and head size or a washer should provide improved compression across the fracture without the head sinking into the metaphyseal cortex.

In the limited available literature, outcomes after surgical fixation of this fracture are excellent with most patients returning to sports within 3 to 6 months of surgical intervention.^9^ There is one report of a limb length discrepancy and compartment syndrome after this injury.^10^ Table 1 summarizes the available literature on this injury. Most cases occur in adolescents between ages 12 through 16 years, a time when growth is rapid and physes are beginning to close.^3,11^ Thus, surgeons may consider following these patients to skeletal maturity to observe for premature physeal closure and angular deformities.

**Table 1:** Summary of the Available Literature on the Triplane Fracture of the Proximal Tibia

Author	Year	Age/Sex	Fixation	Arthroscopic	Complications
Mathews et al	2000	11/F	TE screws	No	None long term
Bos et al	2003	17/M	Staples	Yes	None at 6 mo
Turra et al	2007	15/M	TE screws	No	None at 4 yr
Rabenold et al	2010	11/F	TE screws/pins	No	Compartment syndrome, physeal bar at 1 yr
Dougall et al	2015	14/M	TE screws	No	None at 3 mo
Reilly et al	2017	13/F	TE screws	Yes	None at 6 wk
Abderrazek et al	2019	12/M	TE pins	No	None at 1 yr

## Conclusion

In this article, we add an example of a proximal tibial triplane fracture to the limited literature on this rare injury. Although uncommon, this fracture pattern should be familiar to providers to ensure accurate diagnosis. The fracture can be reliably stabilized with minimally invasive techniques. Cross-sectional imaging can be useful for complete understanding of the fracture pattern and identification of associated injuries. Arthroscopic examination is useful to diagnose and treat additional intra-articular pathology and to confirm anatomic reduction. Long-term follow-up is recommended to evaluate fracture healing and monitor for physeal arrest.
